# Prognostic Significance of End-Stage Liver Diseases, Respiratory Tract Infection, and Chronic Kidney Diseases in Symptomatic Acute Hepatitis E

**DOI:** 10.3389/fcimb.2020.593674

**Published:** 2021-01-15

**Authors:** Huahao Fan, Junfen Fan, Suming Chen, Yangzhen Chen, Huiru Gao, Liying Shan, Xue Li, Fengjun Gu, Hui Zhuang, Lijun Sun

**Affiliations:** ^1^ College of Life Science and Technology, Beijing University of Chemical Technology, Beijing, China; ^2^ Institute of Cerebrovascular Disease Research and Department of Neurology, Xuanwu Hospital of Capital Medical University, Beijing, China; ^3^ The Medical Center of Clinical Laboratory, Beijing 302 Hospital/The Fifth Medical Center of PLA General Hospital, Beijing, China; ^4^ Medical Information Center, Beijing 302 Hospital/The Fifth Medical Center of PLA General Hospital, Beijing, China; ^5^ Department of Microbiology and Infectious Disease Center, School of Basic Medical Sciences, Peking University Health Science Center, Beijing, China; ^6^ Research Center for Clinical and Translational Medicine, Beijing 302 Hospital/The Fifth Medical Center of PLA General Hospital, Beijing, China

**Keywords:** prognostic significance, symptomatic HEV infection, end-stage liver diseases, respiratory tract infection, chronic kidney diseases, clinical outcome

## Abstract

Symptomatic hepatitis E virus (HEV) infection is sporadic, and usually occurs in a limited number of infected patients, which hinders the investigation of risk factors for clinical outcomes in patients with acute HEV infection. A retrospective cohort study enrolling 1913 patients with symptomatic acute hepatitis E in Beijing 302 Hospital from January 1, 2001 to December 31, 2018 was conducted. The baseline characteristics, clinical features and laboratory data of these HEV infection cases were analyzed. Albumin (ALB), platelet (PLT), alanine aminotransferase (ALT), total bilirubin (T-BiL), international normalized ratio (INR) and serum creatinine (SCR) levels, along with the model for end-stage liver disease (MELD) score, hospitalization days, co-morbidity number and mortality were taken as major parameters for comparing the clinical manifestations in our study. We found that not all pre-existing chronic liver diseases exacerbate clinical manifestations of acute hepatitis E. Alcoholic hepatitis, fatty liver hepatitis, hepatic cyst, drug-induced hepatitis and hepatocellular carcinoma were not significantly associated with mortality of HEV patients. Among all of the comorbidities, end-stage liver diseases (ESLDs, including ascites, cirrhosis, hepatic coma and hepatorenal syndrome), respiratory tract infection and chronic kidney diseases (CKDs, including renal insufficiency and renal failure) were found to remarkably increase the mortality of patients with symptomatic HEV infection. Furthermore, the severity evaluation indexes (SEI), such as MELD score, duration of hospital stay, and co-morbidity number in HEV patients with underlying comorbidities were much worse than that of their counterparts without relevant comorbidities.

## Introduction

As the etiological cause of adverse liver events, infection of hepatitis E virus (HEV) has become an important public health problem worldwide, especially in some developing countries with poor sanitary conditions. In 2005, the global cases of HEV infection were estimated to be 20.1 million, and 3.4 million of these were symptomatic cases including 70,000 deaths and 3,000 stillbirths (Rein et al., 2012), and the actual global HEV burden might be worse than this estimate (European Association for the Study of the Liver, 2018). Acute HEV infection can cause severe outcomes, such as symptomatic acute hepatitis, liver failure and even death (Lai et al., 2018). There are still many gaps in our knowledge regarding HEV infection, and the opinions on comorbidities important for the clinical manifestations of HEV patients are controversial. Moreover, current knowledge on the effects of underlying comorbidities to symptomatic acute HEV patients is scarce, and most related clinical studies have been conducted with a limited number of cases (Kumar Acharya et al., 2007; Radha Krishna et al., 2009; Zhang et al., 2010; Zhang et al., 2011; Cheng et al., 2013; Blasco-Perrin et al., 2015; Chen et al., 2016; Zhang et al., 2017; Lai et al., 2018), which hinders the convincing analysis of underlying comorbidities as independent risk factors for symptomatic acute HEV infection. Pre-existing chronic liver diseases, including chronic hepatitis B (CHB) (Lai et al., 2018) and cirrhosis (Kumar Acharya et al., 2007), are commonly considered as risk factors for adverse clinical manifestations of acute hepatitis E (European Association for the Study of the Liver, 2018). However, some other studies showed that chronic hepatitis C (Samala et al., 2016) and alcoholic hepatitis (Haim-Boukobza et al., 2015) are not associated with the poorer manifestation of patients with acute hepatitis E.

Our recent study has demonstrated that it was the background end-stage liver diseases (ESLDs) but not pure hepatitis B virus (HBV) infection that exacerbated the clinical manifestations of patients with acute hepatitis E, implying that ESLDs may play a vital role in affecting the clinical manifestations of acute HEV patients (Sun et al., 2020). There are approximately 257 million chronic HBV patients, of which 887,000 die from HBV related ESLDs every year worldwide (WHO. Global hepatitis report, 2017). Large cohort studies systematically investigating the effects of pre-existing comorbidities, including ESLDs on acute HEV infection, are urgently needed. Furthermore, data from previous studies regarding the effect of co-existing comorbidities on symptomatic acute HEV patients varies, as reported mortality ranging from 0 to 20% (Kumar Acharya et al., 2007; Blasco-Perrin et al., 2015; Chen et al., 2016; Lai et al., 2018). One of the most important reasons for this is a deficiency in the quantity of symptomatic acute HEV patients, especially HEV patients with ESLDs. Here, the clinical features of 1913 anti-HEV IgM antibody positive symptomatic patients were analyzed. ESLDs, respiratory tract infection and chronic kidney diseases (CKDs) were determined to be risk factors for mortality of symptomatic acute HEV patients.

## Methods

### Study Design and Data Source

**Figure 1:**
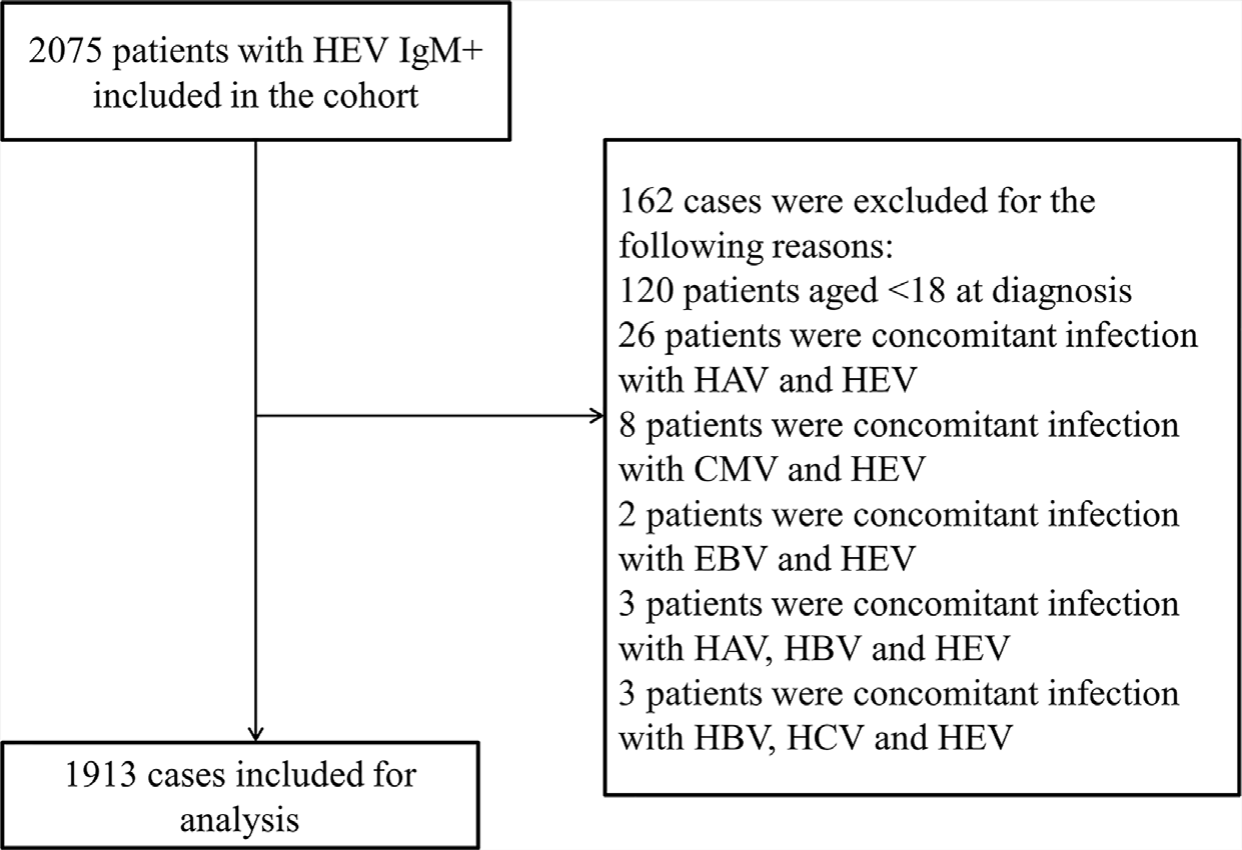
Flowchart of patient inclusions. HAV, hepatitis A virus; HBV, hepatitis B virus; HCV, hepatitis C virus; HEV, hepatitis E virus; IgM^+^, anti-HEV immunoglobulin M; CMV, cytomegalovirus; EBV, Epstein-Barr Virus.

This retrospective registry study was based on data from the Clinical Data Analysis and Reporting System (CDARS) of the Beijing 302 Hospital/The Fifth Medical Center of PLA General Hospital, the largest hepatology tertiary referral hospital in China, where over 95% of patients are with hepatopathy (Huang et al., 2017). The system records all laboratory results and clinicians’ medical records for hospitalized patients. 2075 patients hospitalized with acute HEV infection (anti-HEV immunoglobulin M positive, IgM^+^) between 1 January 2001 and 31 December 2018 were identified. The anti-HEV IgM and IgG antibodies were determined using commercial HEV ELISA Kit (Modern Gaoda, Beijing, China; Registration Certificate Number: CFDA-20133401154, CFDA-20133401615), and the sensitivity and specificity of the anti-HEV IgM kit are 98.5% and 94.8% separately according to the manufacturer’s instructions, the same assay was used throughout the study. 162 cases were excluded by the initial screening due to <18 years old at diagnosis (120 patients) or coexistence of other viral infection markers, including 26 HAV IgM^+^ individuals, 8 CMV IgM^+^ individuals, 2 EBV IgM^+^ individuals, 3 concomitant infection of HAV, HBV and HEV, and 3 concomitant infection of HBV, HCV and HEV individuals. A total of 1913 patients with acute hepatitis E were enrolled in this retrospective study (**Figure 1**).The study was approved by the ethics committee of the Beijing 302 Hospital/The Fifth Medical Center of PLA General Hospital, and the approval number is 2019020D.

### Data Collection

Data were retrieved *via* the hospitals CDARS in April 2019, and baseline date was defined as the date of the first appearance of anti-HEV IgM antibody positivity, and demographic data included sex and age. Information including albumin (ALB) level, blood platelet (PLT) level, alanine aminotransferase (ALT) level, total bilirubin (T-BiL) level, international normalized ratio (INR) level, serum creatinine (SCR) level and comorbidities were collected at baseline. Hospitalization days and mortality were also obtained from the records. All recorded comorbidities including HBV infection, alcoholic hepatitis, ascites, hypertension, cirrhosis, diabetes mellitus, fatty liver hepatitis, hepatic cyst, renal cyst, anemia, biliary cyst, gallstone, cholecystitis, respiratory tract infection, drug-induced hepatitis, hepatocellular carcinoma (HCC), cardiovascular diseases, gallbladder polyp, hepatic coma, renal insufficiency, calculus of kidney, hepatorenal syndrome, hepatic failure, hepatitis C virus (HCV) infection and renal failure were based on ICD-10-CM diagnosis codes and medical records (**Supplementary Table 1**), and all of above information were checked twice by two persons manually according to the records from CDARS.

### Clinical Outcomes

All deaths between January 2001 and December 2018 were determined using data from CDARS. The primary outcome was all-cause mortality, and secondary outcomes included liver-related mortality. Liver-related mortality was defined as death related to hepatic events including hepatic encephalopathy, hepatorenal syndrome and acute liver failure, which were identified based on ICD-10-CM diagnosis codes (**Supplementary Table 1**).

### Definition of Variables

The variables used in the study were identified based on ICD-10-CM diagnosis codes (**Supplementary Table 1**) and are defined as follows:

End-stage liver diseases in our manuscript includes ascites, cirrhosis, hepatic coma and hepatorenal syndrome.

Ascites (ICD-10-CM Code R18) is a gastroenterological term for an accumulation of fluid in the peritoneal cavity. The medical condition is also known as peritoneal cavity fluid, peritoneal fluid excess, hydroperitoneum or more archaically as abdominal dropsy.

Cirrhosis (ICD-10-CM Code K70.3 and K74) is a condition in which the liver does not function properly due to long-term damage. Typically, the disease comes on slowly over months or years. Early on, there are often no symptoms. As the disease worsens, a person may become tired, weak, itchy, have swelling in the lower legs, develop yellow skin, bruise easily, have fluid build up in the abdomen, or develop spider-like blood vessels on the skin. The fluid build-up in the abdomen may become spontaneously infected. Other complications include hepatic encephalopathy, bleeding from dilated veins in the esophagus or dilated stomach veins, and liver cancer. Hepatic encephalopathy results in confusion and possibly unconsciousness.

Hepatic coma (ICD-10-CM Code B19.0) is defined as loss of consciousness due to liver failure. Liver failure occurs because the liver tissue has been irreversibly and progressively destroyed (cirrhosis) as a result of infection, poison, or other disease.

Hepatorenal syndrome (ICD-10-CM Code K76.7) (often abbreviated HRS) is a life-threatening medical condition that consists of rapid deterioration in kidney function in individuals with cirrhosis or fulminant liver failure. HRS is usually fatal unless a liver transplant is performed, although various treatments, such as dialysis, can prevent advancement of the condition.

Respiratory tract infection includes upper respiratory tract infection (ICD-10-CM Code J06) and lower respiratory tract infection (ICD-10-CM Code J22). Upper respiratory infection is an infectious process of any of the components of the upper airway. Infection of the specific areas of the upper respiratory tract can be named specifically. Lower respiratory tract infection (LRTI) is infection below the level of the larynx and may be taken to include bronchiolitis, bronchitis and pneumonia. The presentation of these conditions will depend on age, infecting organism and site of infection.

Chronic kidney diseases (CKDs, ICD-10-CM Code N18) also known as chronic renal disease, is progressive loss in kidney function over a period of months or years. The symptoms of worsening kidney function are not specific, and might include feeling generally unwell and experiencing a reduced appetite.

Renal failure (ICD-10-CM Code N17), also known as kidney failure or renal insufficiency, is a medical condition in which the kidneys fail to adequately filter waste products from the blood. The two main forms are acute kidney injury, which is often reversible with adequate treatment, and chronic kidney disease, which is often not reversible. In both cases, there is usually an underlying cause.

Renal insufficiency is defined as poor function of the kidneys that may be due to a reduction in blood-flow to the kidneys caused by renal artery disease. Normally, the kidneys regulate body fluid and blood pressure, as well as regulate blood chemistry and remove organic waste. Proper kidney function may be disrupted, however, when the arteries that provide the kidneys with blood become narrowed, a condition called renal artery stenosis.

### Statistical Analysis

The model for end-stage liver disease (MELD) scores were calculated based on the formula: MELD=3.78×ln[T-BiL(mg/dl)]+11.2×ln[INR]+9.57×ln[Cr(mg/dl)]+6.43 (Kamath et al., 2001). The T-BiL in the formula was total bilirubin, the INR was the international normalized ratio, Cr was the serum creatinine and ln was the log as the natural logarithm. Data were analyzed using SPSS software version 22.0 (IBM SPSS Statistics). Continuous variables were expressed as mean ± standard deviation (SD) or median (interquartile range) as appropriate, whereas categorical variables were presented as number (percentage). Qualitative and quantitative differences between the two subgroups were analyzed by χ^2^ test or Fisher’s exact test for categorical parameters, and Student *t* test or Mann-Whitney test for continuous parameters as appropriate. Univariate logistic analysis was used for examining the risk factors for the mortality of patients and adjustment logistic analysis was used to do adjustment for age and sex. All statistical tests were 2-sided. Statistical significance was taken as *p*<0.05.

## Results

### Patient Characteristics and Their Clinical Features

From 1 January 2001 to 31 December 2018, 2075 acute symptomatic HEV infected patients were hospitalized in Beijing 302 Hospital/The Fifth Medical Center of PLA General Hospital. In all, 162 cases were excluded because of age (age <18) or other concomitant unexpected viral infections. A total of 1913 patients with acute hepatitis E were enrolled in this retrospective study (**Figure 1**). The number of patients with acute hepatitis E fluctuated every year (**Supplementary Figure 1**), and it seemed that more cases occurred in the first five months than the other months (**Supplementary Figure 2**). The age of the HEV patients was 50.26 ± 13.92 years old, the majority of whom were male (85.80%). The ALB, PLT, ALT, T-BiL, INR and SCR levels, along with the MELD score, hospitalisation days, co-morbidity number, all-cause mortality rate and liver-related mortality rate of the HEV patients were 35.61 ± 8.43 (g/L), 174.80 ± 76.08 (×10^9^ cells/L), 410.00 (100.00,1022.50) (U/L), 125.50 (42.00,243.20) (μmol/L), 1.24 ± 0.70, 82.00 (71.40,93.00) (μmol/L), 15.93 ± 6.62, 27.46 ± 18.82 (days), 2.74 ± 2.43, 2.6% and 2.2%, respectively. The missing rates of the above parameters ranged from 5.0% to 8.6% (**Table 1**).

**Table 1:** Baseline of clinical characteristics.

Characteristic	HEV(n=1913)
Age, y, mean ± SD	50.26 ± 13.92
Male sex	1641.00 (85.80)
ALB, g/L, mean ± SD	35.61 ± 8.43
Missing, %	100 (5.2)
PLT, ×10^9^ cells/L, mean ± SD	174.80 ± 76.08
Missing, %	138 (7.2)
ALT, U/L, median (IQR)	410.00 (100.00,1022.50)
Missing, %	96 (5.0)
T-BiL, μmol/L, median (IQR)	125.50 (42.00,243.20)
Missing, %	97 (5.1)
INR, mean ± SD	1.24 ± 0.70
Missing, %	150 (7.8)
SCR, μmol/L, median (IQR)	82.00 (71.40,93.00)
Missing, %	134 (7.0)
MELD score, mean ± SD	15.93 ± 6.62
Missing, %	165 (8.6)
Hospitalization days	27.46 ± 18.82
Co-morbidity number	2.74 ± 2.43
All-cause mortality	49 (2.6)
Liver-related mortality	42 (2.2)
Diseases	
HBV infection	472 (24.7)
Alcoholic hepatitis	367 (19.2)
Ascites	339 (17.7)
Hypertension	276 (14.4)
Cirrhosis	274 (14.3)
Diabetes mellitus	260 (13.6)
Fatty liver hepatitis	234 (12.2)
Hepatic cyst	201 (10.5)
Renal cyst	193 (10.1)
Anemia	176 (9.2)
Biliary cyst	107 (5.6)
Gallstone	105 (5.5)
Cholecystitis	101 (5.3)
Respiratory tract infection	98 (5.1)
Drug-induced hepatitis	91 (4.8)
HCC	82 (4.3)
Cardiovascular diseases	48 (2.5)
Gallbladder polyp	46 (2.4)
Hepatic coma	34 (1.8)
Renal insufficiency	29 (1.5)
Kidney stone	26 (1.4)
Hepatorenal syndrome	22 (1.2)
Hepatic failure	19 (1.0)
HCV infection	17 (0.9)
Renal failure	11 (0.6)

Data are presented as No. (%) unless otherwise indicated. Percentages are based on non-missing data.

HEV, hepatitis E virus; ALB, albumin; PLT, blood platelet level; ALT, alanine aminotransferase; T-BiL, total bilirubin; INR, international normalized ratio; SCR, serum creatinine; MELD, model for end-stage liver disease; SD, standard deviation; HBV, hepatitis B virus; HCV, hepatitis C virus; HCC, hepatocellular carcinoma.

### Risk Factors for Mortality in Symptomatic HEV-Infected Patients

To elucidate the risk factors for liver-related mortality in symptomatic HEV infected patients, age, gender and all the coexisting comorbidities were incorporated to compare their effects on liver-related mortality in all HEV infected patients and patient subgroups (**Table 2**). And our analysis revealed that end-stage liver diseases [including ascites (OR=14.301, *p*<0.001), cirrhosis (OR=4.731, *p*<0.001), hepatic coma (OR=33.618, *p*<0.001) and hepatorenal syndrome (OR=38.979, *p*<0.001)], respiratory tract infection (OR=11.968, p<0.001), and CKDs [including renal insufficiency (OR=5.459, *p*=0.007), renal failure (OR=42.005, *p*<0.001)] were the significant risk factors of liver-related mortality for HEV patients. And HBV infection was also identified as the risk factors of HEV patients here, but further analysis found it was the background ESLDs, but not pure HBV infection, exacerbated the clinical outcomes of HEV patients (Sun et al., 2020). However, other coexisting comorbidities including alcoholic hepatitis, hypertension, diabetes mellitus, fatty liver hepatitis, hepatic cyst, renal cyst, anemia, gallstone, drug-induced hepatitis, hepatocellular carcinoma, cardiovascular disease, gallbladder polyp, kidney stone, and cholecystitis were not found to be significantly associated with all-cause mortality and liver-related mortality of HEV patients. Notably, after adjusting for both age and sex, ESLDs, respiratory tract infection and CKDs were still identified as risk factors for mortality in symptomatic HEV infected patients (**Table 2**). Furthermore, the liver-related mortality of HEV patients with pre-existing ESLDs (including ascites, cirrhosis, hepatic coma, and hepatorenal syndrome) were significantly higher than that of their counterparts without relevant comorbidities (9.1% vs. 0.7%, *p*<0.001; 6.6% vs. 1.5%, *p*<0.001; 35.3% vs. 1.6%, *p*<0.001; 40.9% vs. 1.7%, *p*<0.001, respectively). And HEV patients with respiratory tract infection had higher liver-related mortality than those without respiratory tract infection (15.3% vs. 1.5%, *p*<0.001). In addition, the liver-related mortality of HEV patients with CKDs (including renal insufficiency and renal failure) was higher than that of HEV patients without relevant CKDs (10.3% vs. 2.1%, *p*=0.024; 45.5% vs. 1.9%, *p*<0.001, respectively) (**Supplementary Table 2**).

**Table 2:** Risk factors for mortality in symptomatic HEV-infected patients.

Characteristic	Univariate Analysis			Adjustment Analysis^a^		
	OR	95%CI	*P* value	aOR	95% CI	*P* value
**All-cause mortality**						
Age	1.018	(0.997–1.040)	0.086	1.019	(0.998–1.040)	0.079
Male sex	1.889	(0.674–5.296)	0.226	1.937	(0.690–5.434)	0.209
End-stage liver diseases						
Ascites	11.586	(6.234–21.533)	<0.001	11.066	(5.906–20.736)	<0.001
Cirrhosis	3.647	(2.010–6.616)	<0.001	3.602	(1.983–6.544)	<0.001
Hepatic coma	31.692	(14.728–68.194)	<0.001	30.153	(13.947–65.193)	<0.001
Hepatorenal syndrome	32.037	(12.950–79.254)	<0.001	27.900	(11.070–70.317)	<0.001
Hepatic failure	NA	NA	NA	NA	NA	NA
Respiratory tract infection	14.310	(7.719–26.529)	<0.001	13.525	(7.149–25.587)	<0.001
Chronic kidney diseases						
Renal insufficiency	4.610	(1.347–15.779)	0.015	3.669	(1.048–12.845)	0.042
Renal failure	51.879	(15.244–176.555)	<0.001	50.290	(14.551–173.806)	<0.001
HBV infection	3.302	(1.867–5.840)	<0.001	3.992	(2.197–7.254)	<0.001
Alcoholic hepatitis	0.947	(0.455–1.968)	0.883	0.857	(0.409–1.796)	0.683
Hypertension	0.520	(0.186–1.458)	0.214	0.418	(0.146–1.198)	0.105
Diabetes mellitus	0.408	(0.126–1.321)	0.135	0.366	(0.112–1.190)	0.095
Fatty liver hepatitis	0.811	(0.318–2.067)	0.661	0.805	(0.315–2.054)	0.650
Hepatic cyst	0.549	(0.169–1.781)	0.318	0.450	(0.137–1.483)	0.189
Renal cyst	0.373	(0.090–1.547)	0.174	0.310	(0.074–1.296)	0.108
Anemia	0.637	(0.196–2.071)	0.454	0.597	(0.183–1.949)	0.392
Biliary cyst	NA	NA	NA	NA	NA	NA
Gallstone	0.728	(0.174–3.037)	0.663	0.625	(0.148–2.634)	0.522
Drug-induced hepatitis	1.316	(0.401–4.315)	0.650	1.442	(0.434–4.787)	0.550
HCC	0.949	(0.226–3.976)	0.943	0.899	(0.214–3.770)	0.884
Cardiovascular diseases	1.682	(0.396–7.134)	0.481	1.344	(0.308–5.865)	0.694
Gallbladder polyp	1.760	(0.414–7.476)	0.444	1.744	(0.410–7.426)	0.452
Kidney stone	1.532	(0.203–11.543)	0.679	1.408	(0.186–10.635)	0.740
Cholecystitis	0.759	(0.182–3.169)	0.705	0.665	(0.158–2.794)	0.578
HCV infection	NA	NA	NA	NA	NA	NA
**Liver-related mortality**						
Age	1.024	(1.001–1.048)	0.038	1.025	(1.002–1.048)	0.034
Male sex	2.183	(0.670–7.114)	0.195	2.258	(0.692–7.368)	0.177
End-stage liver diseases						
Ascites	14.301	(7.112–28.760)	<0.001	13.374	(6.597–27.115)	<0.001
Cirrhosis	4.731	(2.532–8.841)	<0.001	4.704	(2.511–8.811)	<0.001
Hepatic coma	33.618	(15.250–74.110)	<0.001	31.811	(14.321–70.663)	<0.001
Hepatorenal syndrome	38.979	(15.581–97.512)	<0.001	32.602	(12.769–83.244)	<0.001
Hepatic failure	NA	NA	NA	NA	NA	NA
Respiratory tract infection	11.968	(6.134–23.351)	<0.001	10.688	(5.373–21.262)	<0.001
Chronic kidney diseases						
Renal insufficiency	5.459	(1.585–18.796)	0.007	4.081	(1.156–14.413)	0.029
Renal failure	42.005	(12.270–143.796)	<0.001	40.421	(11.521–141.813)	<0.001
HBV infection	3.474	(1.878–6.423)	<0.001	4.420	(2.316–8.435)	<0.001
Alcoholic hepatitis	0.991	(0.455–2.159)	0.982	0.889	(0.405–1.953)	0.770
Hypertension	0.450	(0.138–1.467)	0.186	0.342	(0.103–1.137)	0.080
Diabetes mellitus	0.483	(0.148–1.575)	0.228	0.423	(0.129–1.387)	0.156
Fatty liver hepatitis	0.751	(0.266–2.124)	0.589	0.747	(0.264–2.116)	0.583
Hepatic cyst	0.650	(0.199–2.123)	0.476	0.508	(0.153–1.688)	0.269
Renal cyst	0.440	(0.105–1.834)	0.260	0.350	(0.083–1.474)	0.152
Anemia	0.488	(0.117–2.035)	0.325	0.444	(0.106–1.864)	0.267
Biliary cyst	NA	NA	NA	NA	NA	NA
Gallstone	0.858	(0.205–3.601)	0.835	0.707	(0.166–3.000)	0.638
Drug-induced hepatitis	1.559	(0.472–5.142)	0.466	1.733	(0.518–5.800)	0.372
HCC	0.539	(0.073–3.967)	0.544	0.507	(0.069–3.737)	0.505
Cardiovascular diseases	1.984	(0.465–8.456)	0.355	1.484	(0.337–6.532)	0.602
Gallbladder polyp	2.076	(0.486–8.862)	0.324	2.061	(0.481–8.830)	0.330
Kidney stone	NA	NA	NA	NA	NA	NA
Cholecystitis	0.895	(0.213–3.757)	0.879	0.759	(0.180–3.208)	0.708
HCV infection	NA	NA	NA	NA	NA	NA

OR, odds ratio; aOR, adjusted odds ratio; CI, confidence interval; HEV, hepatitis E virus; HCV, hepatitis C virus; HCC, hepatocellular carcinoma; NA, not available. **^a^** Adjustment model was adjusted for both age and sex.

### End-Stage Liver Diseases Were Associated With More Severe Outcomes in Patients With Acute Hepatitis E

A total of 473 HEV patients (24.7%) presented with ESLDs, and the HEV/ESLDs patients were older than HEV patients without ESLDs (52.48 ± 12.89 vs. 49.53 ± 14.17, *p*<0.001) (**Table 3**). The male proportion of the HEV/ESLDs patients was higher than HEV patients without ESLDs (90.7% vs. 84.2%, *p*<0.001). The ALB, PLT and ALT levels of the HEV/ESLDs patients were lower than in those of HEV patients without ESLDs [32.33 ± 6.10 vs. 36.71 ± 8.82, *p*<0.001; 124.48 ± 67.90 vs. 192.04 ± 70.93, *p*<0.001; 182.00 (65.50,689.50) vs. 517.00 (131.00,1145.00), *p*<0.001, respectively]. In contrast, the T-BiL, INR and SCR levels of the HEV/ESLDs patients were much higher than in those of HEV patients without ESLDs [245.20(124.00,387.95) vs. 99.20(34.65,188.88), *p*<0.001; 1.62 ± 0.84 vs. 1.11 ± 0.60, *p*<0.001; 85.50 (73.00,103.75) vs. 81.00 (71.00,91.00), *p*<0.001, respectively]. Severity evaluation indexes (SEI), including the MELD score, hospitalization days, co-morbidity number, all-cause mortality rate and liver-related mortality rate of HEV patients with ESLDs, were significantly poorer than those in the patients without ESLDs (21.26 ± 7.43 vs. 14.10 ± 5.18, *p*<0.001; 33.31 ± 24.26 vs. 25.54 ± 16.21, *p*<0.001; 3.60 ± 1.57 vs. 1.34 ± 1.25, *p*<0.001; 8.0% vs. 0.8%, *p*<0.001, 7.4% vs. 0.5%, *p*<0.001, respectively) (**Table 3**).

**Table 3:** Demographic and clinical characteristics of HEV patients with or without end-stage liver diseases.

Characteristic	HEV without end-stage liver diseases (n=1440)	HEV with end-stage liver diseases (n=473)	*P* value
Age, y, mean ± SD	49.53 ± 14.17	52.48 ± 12.89	<0.001
Male sex	1212(84.2)	429(90.7)	<0.001
ALB, g/L, mean ± SD	36.71 ± 8.82	32.33 ± 6.10	<0.001
Missing, %	84(5.8)	16(3.4)	
PLT, ×10^9^ cells/L, mean ± SD	192.04 ± 70.93	124.48 ± 67.90	<0.001
Missing, %	118(8.2)	20(4.2)	
ALT, U/L, median (IQR)	517.00(131.00,1145.00)	182.00(65.50,689.50)	<0.001
Missing, %	80(5.6)	16(3.4)	
T-BiL, μmol/L, median (IQR)	99.20(34.65,188.88)	245.20(124.00,387.95)	<0.001
Missing, %	80(5.6)	17(3.6)	
INR, mean ± SD	1.11 ± 0.60	1.62 ± 0.84	<0.001
Missing, %	128(8.9)	22(4.6)	
SCR, μmol/L, median (IQR)	81.00(71.00,91.00)	85.50(73.00,103.75)	<0.001
Missing, %	113(7.8)	21(4.4)	
MELD score, mean ± SD	14.10 ± 5.18	21.26 ± 7.43	<0.001
Missing, %	140(9.7)	25(5.3)	
Hospitalization days	25.54 ± 16.21	33.31 ± 24.26	<0.001
Co-morbidity number	1.34 ± 1.25	3.60 ± 1.57	<0.001
All-cause Mortality	11(0.8)	38(8.0)	<0.001
Liver-related mortality	7(0.5)	35(7.4)	<0.001

HEV, hepatitis E virus; ALB, albumin; PLT, blood platelet level; ALT, alanine aminotransferase; T-BiL, total bilirubin; INR, international normalized ratio; SCR, serum creatinine; MELD, Model for End-Stage Liver Disease; SD, standard deviation.

These acute HEV patients with ESLDs included patients with ascites (339 cases), cirrhosis (274 cases), hepatic coma (34 cases), and hepatorenal syndrome (22 cases). And the effect of specific ESLDs including ascites, cirrhosis, hepatic coma, and hepatorenal syndrome on the clinical manifestations of HEV patients were evaluated. The SEI, including MELD score, hospitalization days, co-morbidity number and mortality of HEV/specific ESLD were significantly more severe than in those of the HEV patients without specific ESLD (**Supplementary Tables 3–6**).

### Respiratory Tract Infection Was Associated With Poorer Outcomes in HEV Patients

Among the 1913 acute HEV patients, 98 (5.1%) presented with respiratory tract infection (**Table 4**). The HEV/respiratory tract infected patients were older than HEV patients without respiratory tract infection (57.33 ± 12.39 vs. 49.88 ± 13.90, *p*<0.001). There was no significant male proportional difference between HEV patients with respiratory tract infection and those without respiratory tract infection (89.8% vs. 85.6%, *p*=0.243). The ALB, PLT and ALT levels of the HEV/respiratory tract infected patients were lower than in those of HEV patients without respiratory tract infection [31.63 ± 6.62 vs. 35.83 ± 8.47, *p*<0.001; 140.37 ± 80.15 vs. 176.73 ± 75.41, *p*<0.001; 166.00(54.50,570.50) vs. 433.00(105.00,1048.00), *p*<0.001, respectively]. In contrast, the T-BiL, INR and SCR levels of HEV/respiratory tract infected patients were much higher than in those of HEV patients without respiratory tract infection [248.15 (139.05,395.25) vs. 120.05 (39.78,233.30), *p*<0.001; 1.58 ± 0.94 vs. 1.22 ± 0.68, *p*<0.001; 87.50 (73.00,110.75) vs. 82.00 (71.00,92.00), *p*=0.003, respectively]. Hence, the MELD score of the HEV/respiratory tract infected patients was significantly higher than that of HEV patients without respiratory tract infection (21.72 ± 7.01 vs. 15.60 ± 6.45, *p*<0.001). Consistently, the SEI, including hospitalization days, co-morbidity number, all-cause mortality rate and liver-related mortality rate of HEV/respiratory tract infected patients, were poorer than that of HEV patients without respiratory tract infection (33.61 ± 26.76 vs. 27.13 ± 18.24, *p*<0.001; 3.77 ± 1.78 vs. 1.80 ± 1.59, *p*<0.001; 19.4% vs. 1.7%, *p*<0.001, 15.3% vs. 1.5%, *p*<0.001, respectively) (**Table 4**).

**Table 4:** Demographic and clinical characteristics of HEV patients with or without respiratory tract infection.

Characteristic	HEV without respiratory tract infection (n=1815)	HEV with respiratory tract infection (n=98)	*P* value
Age, y, mean ± SD	49.88 ± 13.90	57.33 ± 12.39	<0.001
Male sex	1553(85.6)	88(89.8)	0.243
ALB, g/L, mean ± SD	35.83 ± 8.47	31.63 ± 6.62	<0.001
Missing, %	96(5.3)	4(4.1)	
PLT, ×10^9^ cells/L, mean ± SD	176.73 ± 75.41	140.37 ± 80.15	<0.001
Missing, %	134(7.4)	4(4.1)	
ALT, U/L, median (IQR)	433.00(105.00,1048.00)	166.00(54.50,570.50)	<0.001
Missing, %	92(5.1)	4(4.1)	
T-BiL, μmol/L, median (IQR)	120.05(39.78,233.30)	248.15(139.05,395.25)	<0.001
Missing, %	93(5.1)	4(4.1)	
INR, mean ± SD	1.22 ± 0.68	1.58 ± 0.94	<0.001
Missing, %	146(8.0)	4(4.1)	
SCR, μmol/L, median (IQR)	82.00(71.00,92.00)	87.50(73.00,110.75)	0.003
Missing, %	130(7.2)	4(4.1)	
MELD score, mean ± SD	15.60 ± 6.45	21.72 ± 7.01	<0.001
Missing, %	161(8.9)	4(4.1)	
Hospitalization days	27.13 ± 18.24	33.61 ± 26.76	<0.001
Co-morbidity number	1.80 ± 1.59	3.77 ± 1.78	<0.001
All-cause mortality	30(1.7)	19(19.4)	<0.001
Liver-related mortality	27(1.5)	15(15.3)	<0.001

HEV, hepatitis E virus; ALB, albumin; PLT, blood platelet level; ALT, alanine aminotransferase; T-BiL, total bilirubin; INR, international normalized ratio; SCR, serum creatinine; MELD, Model for End-Stage Liver Disease; SD, standard deviation.

### Chronic Kidney Diseases Deteriorate Clinical Outcomes in Patients With Acute Hepatitis E

A total of 40 symptomatic HEV patients (2.1%) presented with CKDs, and the HEV/CKDs patients were older than HEV patients without CKDs (59.15 ± 12.41 vs. 50.07 ± 13.89, *p*<0.001) (**Table 5**). The ALB, PLT and ALT levels of the HEV/CKDs patients were lower than in those of HEV patients without CKDs [29.00 ± 4.42 vs. 35.76 ± 8.44, *p*<0.001; 135.71 ± 68.20 vs. 175.68 ± 76.04, *p*<0.001; 115.00 (53.00,459.00) vs. 421.00 (103.00,1026.25), *p*=0.012, respectively]. The T-BiL, INR and SCR levels of the HEV/CKDs patients were much higher than that in HEV patients without CKDs [261.40 (109.40, 436.80) vs. 123.60 (41.70, 239.45), *p*<0.001; 1.48 ± 0.67 vs. 1.24 ± 0.70, *p*=0.030; 145.00 (111.00, 246.00) vs. 82.00 (71.00, 92.00), *p*<0.001, respectively]. The SEI, including MELD score, co-morbidity number, all-cause mortality and liver-related mortality of the HEV patients with CKDs were more severe than in those of HEV patients without CKDs (25.20 ± 6.41 vs. 15.72 ± 6.48, *p*<0.001; 4.28 ± 1.68 vs. 1.85 ± 1.62, *p*<0.001; 22.5% vs. 2.1%, *p*<0.001, 20.0% vs. 1.8%, *p*<0.001, respectively) (**Table 5**). And the effect of specific CKD including renal insufficiency and renal failure on the clinical manifestations of HEV patients were evaluated. The SEI, including MELD score, co-morbidity number, all-cause mortality rate and liver-related mortality rate of HEV/specific CKD were significantly more severe than in those of the HEV patients without specific CKD (**Supplementary Tables 7–8**).

**Table 5:** Demographic and clinical characteristics of HEV patients with or without chronic kidney diseases.

Characteristic	HEV without CKDs (n = 1873)	HEV with CKDs (n = 40)	*P* value
Age, y, mean ± SD	50.07 ± 13.89	59.15 ± 12.41	<0.001
Male sex	1603 (85.6)	38 (95.0)	0.092
ALB, g/L, mean ± SD	35.76 ± 8.44	29.00 ± 4.42	<0.001
Missing, %	99 (5.3)	1 (2.5)	
PLT, ×10^9^cells/L, mean ± SD	175.68 ± 76.04	135.71 ± 68.20	0.001
Missing, %	137 (7.3)	1 (2.5)	
ALT, U/L, median (IQR)	421.00 (103.00–1026.25)	115.00 (53.00–459.00)	0.012
Missing, %	95 (5.1)	1 (2.5)	
T-BiL, μmol/L, median (IQR)	123.60 (41.70–239.45)	261.4 0(109.40–436.80)	<0.001
Missing, %	96 (5.1)	1 (2.5)	
INR, mean ± SD	1.24 ± 0.70	1.48 ± 0.67	0.030
Missing, %	149 (7.9)	1 (2.5)	
SCR, μmol/L, median (IQR)	82.00 (71.00–92.00)	145.00 (111.00–246.00)	<0.001
Missing, %	133 (7.1)	1 (2.5)	
MELD score, mean ± SD	15.72 ± 6.48	25.20 ± 6.41	<0.001
Missing,%	164 (8.8)	1 (2.5)	
Hospitalization days	27.41 ± 18.71	29.78 ± 23.67	0.425
Co-morbidity number	1.85 ± 1.62	4.28 ± 1.68	<0.001
All-cause Mortality	40 (2.1)	9 (22.5)	<0.001
Liver-related mortality	34 (1.8)	8 (20.0)	<0.001

HEV, hepatitis E virus; ALB, albumin; PLT, blood platelet level; ALT, alanine aminotransferase; T-BiL, total bilirubin; INR, international normalized ratio; SCR, serum creatinine; MELD, Model for End-Stage Liver Disease; SD, standard deviation.

## Discussion

HEV infection is one of the major causes of acute viral hepatitis worldwide (European Association for the Study of the Liver, 2018; Webb and Dalton, 2019), the transmission routes of HEV vary among four different genotypes, two of them (gt 1 and 2) only infect humans mainly by contaminated drinking water, and gt 3 and 4 are considered as zoonotic infections and have been isolated from humans, pigs, deer, wild boars and other animals and primarily transmitted by pork consumption (Wedemeyer et al., 2012). Compared with gt 1 and 2, gt 3 and 4 are widespread in endemic area mainly in the industrialized countries (Bihl and Negro, 2010). Gt 3, the predominant genotype in Europe and the USA (Horvatits et al., 2018), is usually asymptomatic in people with normal immune function, while it can develop into chronic disease in immunocompromised patients (Sayed et al., 2016), gt 4 is thought to be more pathogenic than gt 1 and 2 (Jeblaoui et al., 2013), and the predominant genotype in China is supposed to change from gt 1 to gt 4 (Liu et al., 2012; Dai et al., 2013; Huang et al., 2014; Kamar et al., 2017; Dalton and Izopet, 2018; Wang et al., 2018), and the majority of HEV infection cases in our study are supposed to be caused by gt 4 HEV virus.

When comparing the effects of comorbidities on the clinical manifestations of HEV patients, it is necessary to have a sufficient number of cases in the cohort study to guarantee a reliable conclusion. Our findings are based on the largest-scale HEV retrospective study to date, containing more symptomatic HEV cases than ever reported, making it possible to better investigate the comorbidities that affect the clinical manifestations of acute HEV patients (Kumar Acharya et al., 2007; Radha Krishna et al., 2009; Zhang et al., 2010; Zhang et al., 2011; Cheng et al., 2013; Blasco-Perrin et al., 2015; Chen et al., 2016; Zhang et al., 2017; Lai et al., 2018). ALB, PLT, ALT, T-BiL, INR and SCR levels, along with the MELD score, hospitalization days, co-morbidity number, all-cause mortality and liver-related mortality were taken as major parameters for comparing the clinical manifestations in our study. As many as 25 comorbidities recorded in the system were used to record the co-morbidity number and to determine the underlying risk factors of the HEV infection. Among these comorbidities, ESLDs, respiratory tract infection and CKDs were determined as significant mortality risk factors for symptomatic patients with acute hepatitis E.

Studies from different groups have reached different conclusions on the effect of cirrhosis or other end-stage liver diseases on patients with acute hepatitis E. A multi-center study performed in the United Kingdom and France showed that hepatitis E infection had no impact on the mortality of decompensated chronic liver disease, and only 11 of 343 cirrhotic patients (3.2%) had acute HEV infections (Blasco-Perrin et al., 2015). However, a Chinese retrospective study demonstrated that the existence of cirrhosis led to poorer outcomes in 228 HBV/HEV superinfected patients (Chen et al., 2016), and another study from India revealed that acute HEV infection increased the mortality of cirrhotic patients to 20% (Kumar Acharya et al., 2007). The above studies’ differing conclusions could be due to a limited number of HEV infection cases, and the findings from our study on the effect of ESLDs in HEV patients were based on the largest cohort of acute HEV patients to date (Kumar Acharya et al., 2007; Radha Krishna et al., 2009; Zhang et al., 2010; Cheng et al., 2013; Chow et al., 2014; Chen et al., 2016; Zhang et al., 2017; Lai et al., 2018). This guarantees the accuracy and reliability of the conclusion of our study. We further dissected the effect of specific ESLD on the clinical manifestations of HEV patients, and ESLDs, including ascites, cirrhosis, hepatic coma and hepatorenal syndrome, were found to exacerbate the clinical manifestations of acute HEV patients. Additionally, we also found that ESLDs progressively worsened the clinical manifestations of HBV/HEV superinfected patients (**Supplementary Table 9**).

As a previously speculated risk factor for acute HEV patients, renal failure was confirmed as an important predictor for adverse clinical features of HEV patients in our study (Zhang et al., 2017; Lai et al., 2018). Most of the parameters suggested that renal failure was an extremely dangerous co-morbidity in patients with acute hepatitis E. And in our study, the hospitalization days of patients with HEV/renal failure were shorter than HEV patients without renal failure, this was probably due to the fact that more than half of the patients with HEV/renal failure suffered from severe illness and death, which shortened the hospitalization days (**Supplementary Tables 7–8**). Besides renal failure, another chronic kidney disease, renal insufficiency, was found to be associated with the adverse clinical manifestations of acute HEV patients. Co-existing CKDs were shown to be associated with poorer clinical manifestations in acute HEV patients, probably because the patients with CKDs were at a higher risk of platelet dysfunction, which led to higher bleeding risks and infections by other pathogens (e.g. pneumonia) (Boccardo et al., 2004; Dalrymple and Go, 2008; Kurts et al., 2013; McDonald et al., 2014). Coincidentally, respiratory tract infection was another identified risk factor of acute HEV patients. The 98 HEV patients (5.1%), presenting with respiratory tract infection, showed more severe clinical features than those of HEV patients without respiratory tract infection, based on multiple parameters. Respiratory infections were found to be independent predictors of acute or acute-on-chronic liver failure (A(C)LF) in patients with hepatitis E (Wang et al., 2019). Besides, the study by Zhang et al. identified pre-existing extrahepatic tumors, diabetes, and chronic respiratory and renal diseases as novel independent predictors for adverse clinical outcomes of symptomatic human hepatitis E virus infection (Zhang et al., 2017). It is interesting that respiratory tract infection is a risk factor of acute HEV patients, and this may be due to the fact that patients with acute HEV infection, especially the severe cases usually have poor immunity, which also makes them more susceptible to respiratory pathogens, further aggravates the symptoms caused by HEV virus. Furthermore, studies have shown that respiratory infection increases serum aminotransferase (ALT and AST) levels and causes liver histopathologic injury (Zhang et al., 2019). Respiratory infections elicit robust hepatic transcriptome remodeling within hours of experimentally induced pneumonia (Quinton et al., 2009). The functional relevance and mechanisms governing respiratory infection and liver function need to be further evaluated. In the future, special attention should be paid to acute HEV patients with respiratory tract infection.

The HEV vaccine is available and currently licensed in China, and phase III clinical trials have shown that the vaccine is 100% effective against symptomatic hepatitis E. As such, it seems that HEV will become a vaccine-preventable disease (Zhu et al., 2010; Teshale and Ward, 2015; Zhang et al., 2015). However, more than 97% of HEV infections are subclinical and asymptomatic (Huang et al., 2014; Lhomme et al., 2019), and because of the relative rarity of symptomatic cases, universal HEV vaccination may be considered not to be cost-effective (Huang et al., 2014; Zhang et al., 2014; Zhu et al., 2014; Zhang et al., 2015). Although most HEV infections are asymptomatic, the clinical manifestations of HEV patients with ESLDs, respiratory tract infection or CKDs seem to cause extremely adverse effects in our study. A total of 473, 98 and 40 out of 1913 symptomatic HEV patients had ESLDs, respiratory tract infection and CKDs, respectively. In light of ESLDs and CKDs being not uncommon among symptomatic HEV patients, and considering their adverse impact on the clinical features of HEV patients, appropriate measures should be taken (especially in high-risk groups, e.g., patients with ESLDs and CKDs), including raising public awareness of the higher-than-expected incidence and hazards of HEV infection and alerting the high-risk population for HEV vaccination.

There are several limitations in our study. First, the specific HEV genotype for each infected patient in our retrospective study was not determined since the HEV genotyping is not a routine examination. However, with large sample size and clinical features (e.g., baseline characteristics, clinical features and laboratory data of the infection cases) as well as outcomes (e.g., the severity evaluation indexes (SEI), such as MELD score, hospitalization days, comorbidity number and mortality) were systematically analyzed, we believe our research give important prognostic significance for end-stage liver diseases, respiratory tract infection and chronic kidney diseases in symptomatic acute Hepatitis E. Second, it was a retrospective study with data collected from only one local hepatology tertiary referral hospital. The timing and frequency of blood tests were heterogeneous. However, the use of clinical outcomes has minimized the effect of this limitation. Third, there were missing data from the cohort, which might limit the statistical power of these subgroups. Moreover, some patients with mild acute hepatitis E who were not hospitalized would have been missed. In addition, we no doubt missed patients who were asymptomatic.

Pre-existing chronic liver diseases are speculated as risk factors of HEV infection, however, current opinions on which comorbidities are important for the clinical manifestations of HEV patients are controversial (European Association for the Study of the Liver, 2018). Here, pre-existing chronic diseases including ESLDs, respiratory tract infection and CKDs but not alcoholic hepatitis, hypertension, fatty liver hepatitis, diabetes mellitus, anemia, drug-induced hepatitis or liver cancer are confirmed as mortality risk factors of acute hepatitis E, based on our largest-scale HEV retrospective study to date. Our study is of significance to highlight several important comorbidities, including ESLDs, respiratory tract infection and CKDs, as risk factors for adverse clinical features of acute HEV patients, and to highlight prophylactic HEV vaccination as urgent measure for relevant at-risk populations. Future studies on the effect of HEV vaccine on high-risk patients with ESLDs, respiratory tract infection and CKDs will be warranted. In conclusion, our study not only greatly enriches people’s understanding of HEV (especially gt 4), but also helps to guide the prevention and treatment of HEV infection.

## Data Availability Statement

The original contributions presented in the study are included in the article/**Supplementary Material**. Further inquiries can be directed to the corresponding authors.

## Ethics Statement

The studies involving human participants were reviewed and approved by by the ethics committee of the Beijing 302 Hospital/The Fifth Medical Center of PLA General Hospital, and the approval number is 2019020D. The patients/ participants provided their written informed consent to participate in this study. Written informed consent was obtained from the individual(s) for the publication of any potentially identifiable images or data included in this article.

## Author Contributions

HZ, HF, and LSu designed the study. HF, JF, SC, YC, HG, LSh, XL, and FG collected the clinical and laboratory data. HZ, HF, and LSu carried out the analysis and interpretation of data. HF drafted the manuscript. HF, YC, HZ and LSu reviewed the manuscript. All authors contributed to the article and approved the submitted version.

## Funding

This research was supported by grants from the National Key Research and Development Program of China (2020YFA0712100), Fundamental Research Funds for Central Universities (BUCTZY2022), the National Natural Science Foundation of China (82001268).

## Conflict of Interest

The authors declare that the research was conducted in the absence of any commercial or financial relationships that could be construed as a potential conflict of interest.
