# Supplementary material for: Prognostic Significance of End-Stage Liver Diseases, Respiratory Tract Infection, and Chronic Kidney Diseases in Symptomatic Acute Hepatitis E
Source: Front Cell Infect Microbiol. 2021 Jan 15;10:593674. doi: 10.3389/fcimb.2020.593674 (PMC7843426; doi:10.3389/fcimb.2020.593674)
Supplement: Supplementary file 1 [file DataSheet_1.doc]

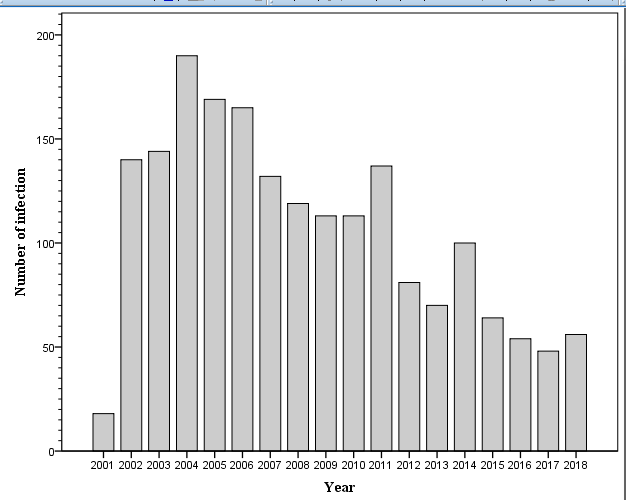


**Supplementary Figure 1.** New HEV case numbers by year from 2001 to 2018 in the study.


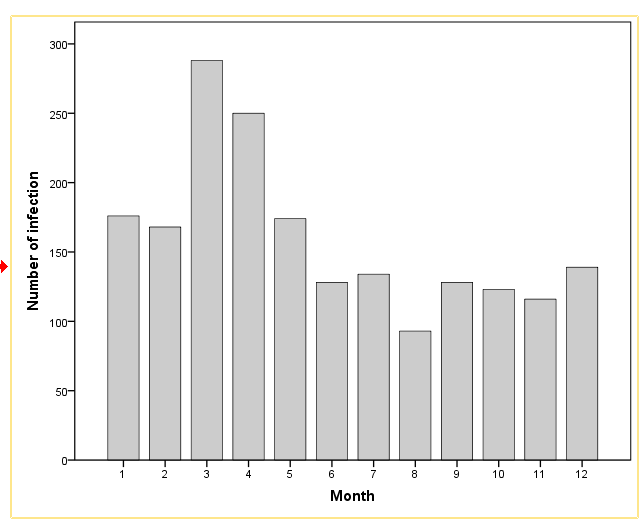


**Supplementary Figure 2.** New HEV case numbers of different months from 2001 to 2018 in the study.

**Supplementary Table 1.** **Diagnostic codes for diseases in patients with symptomatic acute hepatitis E.**

| **Disease** | **Code** | **Description** |
| --- | --- | --- |
| Acute respiratory infections | J06 | Acute upper respiratory infections of multiple and unspecified sites |
| Acute respiratory infections | J22 | Unspecified acute lower respiratory infection |
| Alcoholic fatty liver | K70.0 | Alcoholic fatty liver |
| Alcoholic hepatitis | K70.1 | Alcoholic hepatitis |
| Anemia | D50 | Iron deficiency anemia |
| Ascites | R18 | Ascites |
| Biliary cyst | K83.5 | Biliary cyst |
| Calculus of kidney | N20.0 | Calculus of kidney |
| Cardiovascular diseases | P29 | Cardiovascular disorders originating in the perinatal period |
| Cholecystitis | K81 | Cholecystitis |
| Chronic kidney disease | N18 | Chronic kidney disease |
| Chronic viral hepatitis | B18 | Chronic viral hepatitis |
| Cirrhosis | K70.3 | Alcoholic cirrhosis of liver |
| Cirrhosis | K74 | Fibrosis and cirrhosis of liver |
| Cirrhosis | K74.5 | Biliary cirrhosis, unspecified |
| Coronary atherosclerosis | I25.1 | Atherosclerotic heart disease of native coronary artery |
| Diabetes mellitus | E10 | Type 1 diabetes mellitus |
| Diabetes mellitus | E11 | Type 2 diabetes mellitus |
| Diabetes mellitus | E13 | Other specified diabetes mellitus |
| Diabetes mellitus | E08 | Diabetes mellitus due to underlying condition |
| Diabetes mellitus | E09 | Drug or chemical induced diabetes mellitus |
| Drug-induced hepatitis | K71.6 | Toxic liver disease with hepatitis, not elsewhere classified |
| Fatty liver | K76 | Fatty (change of) liver, not elsewhere classified |
| Gallbladder polyps | K82 | Other disorders of gallbladder |
| Gallstone | K56.3 | Gallstone ileus |
| Hepatic coma | B19.0 | Unspecified viral hepatitis with hepatic coma |
| Hepatic cyst | Q44.6 | Cystic disease of liver |
| Hepatic failure | K72 | Hepatic failure, not elsewhere classified |
| Hepatocellular carcinoma | C22 | Malignant neoplasm of liver and intrahepatic bile ducts |
| Hepatocellular carcinoma | C78.7 | Secondary malignant neoplasm of liver and intrahepatic bile duct |
| Hepatorenal syndrome | K76.7 | Hepatorenal syndrome |
| Hypertension | I10 | Essential (primary) hypertension |
| Kidney/Renal failure | N19 | Unspecified kidney failure |
| Kidney/Renal failure | N17 | Acute kidney failure |
| Renal calculus/stone | N20.0 | Calculus of kidney |
| Renal cyst | N28.1 | Cyst of kidney, acquired |
| Renal failure | N17 | Acute kidney failure |

**Supplementary Table 2. The effect of co-morbidities on mortality of patients with hepatitis E virus.**

| **Co-morbidity** | **HEV without comorbidity** | **HEV with comorbidity** | | ***P* value** |
| --- | --- | --- | --- | --- |
| **All-cause mortality** |  |  | |  |
| End-stage liver diseases | 11(0.8) | | 38(8.0) | <0.001 |
| Ascites | 15(1.0) | | 34(10.0) | <0.001 |
| Cirrhosis | 31(1.9) | | 18(6.6) | <0.001 |
| Hepatic coma | 36(1.9) | | 13(38.2) | <0.001 |
| Hepatorenal syndrome | 40(2.1) | | 9(40.9) | <0.001 |
| Hepatic failure | 49(2.6) | | 0(0.0) | 0.478 |
| Respiratory tract infection | 30(1.6) | | 19(19.4) | <0.001 |
| Chronic kidney diseases | 40(2.1) | | 9(22.5) | <0.001 |
| Renal insufficiency | 46(2.4) | | 3(10.3) | 0.036 |
| Renal failure | 43(2.3) | | 6(54.5) | <0.001 |
| **Liver-related mortality** |  | |  |  |
| End-stage liver diseases | 7(0.5) | | 35(7.4) | <0.001 |
| Ascites | 11(0.7) | | 31(9.1) | <0.001 |
| Cirrhosis | 24(1.5) | | 18(6.6) | <0.001 |
| Hepatic coma | 30(1.6) | | 12(35.3) | <0.001 |
| Hepatorenal syndrome | 33(1.7) | | 9(40.9) | <0.001 |
| Hepatic failure | 42(2.2) | | 0(0.0) | 0.512 |
| Respiratory tract infection | 27(1.5) | | 15(15.3) | <0.001 |
| Chronic kidney diseases | 34(1.8) | | 8(20.0) | <0.001 |
| Renal insufficiency | 39(2.1) | | 3(10.3) | 0.024 |
| Renal failure | 37(1.9) | | 5(45.5) | <0.001 |

Data are presented as No. (%) unless otherwise indicated. *P* value were estimated with χ2 test or Fisher exact test, whenever appropriate.

**Supplementary Table 3. Demographic and clinical characteristics of HEV patients with or without ascites.**

| **Characteristic** | **HEV without ascites (n=1574)** | **HEV with ascites**  **(n=339)** | ***P* value** |
| --- | --- | --- | --- |
| Age, y, mean ± SD | 49.74±14.00 | 52.68±13.26 | <0.001 |
| Male sex | 1325(84.2) | 316(93.2) | <0.001 |
| ALB, g/L, mean ± SD | 36.48±8.70 | 31.69±5.64 | <0.001 |
| Missing, % | 85(5.7) | 11(3.2) |  |
| PLT, ×10^9^ cells/L, mean ± SD | 186.16±73.11 | 124.51±68.30 | <0.001 |
| Missing, % | 126(8.0) | 12(3.5) |  |
| ALT, U/L, median (IQR) | 499.00(115.00,1116.50) | 180.50(68.00,651.75) | <0.001 |
| Missing, % | 85(5.6) | 11(3.2) |  |
| T-BiL, μmol/L, median (IQR) | 102.60(34.70,194.55) | 275.20(171.00,414.80) | <0.001 |
| Missing, % | 85(5.7) | 12(3.5) |  |
| INR, mean ± SD | 1.14±0.63 | 1.68±0.84 | <0.001 |
| Missing, % | 136(8.6) | 14(4.1) |  |
| SCR, μmol/L, median (IQR) | 81.00(71.00,91.00) | 88.00(76.00,106.25) | <0.001 |
| Missing, % | 121(7.7) | 13(3.8) |  |
| MELD score, mean ± SD | 14.40±5.51 | 22.68±6.91 | <0.001 |
| Missing, % | 149(9.5) | 16(4.7) |  |
| Hospitalization days | 25.88±16.86 | 34.83±24.80 | <0.001 |
| Co-morbidity number | 1.49±1.35 | 3.79±1.62 | <0.001 |
| All-cause mortality | 15(1.0) | 34(10.0) | <0.001 |
| Liver-related mortality | 11(0.7) | 31(9.1) | <0.001 |

Abbreviations: HEV, hepatitis E virus; ALB, albumin; PLT, blood platelet level; ALT, alanine aminotransferase; T-BiL, total bilirubin; INR, international normalized ratio; SCR, serum creatinine; MELD, Model for End-Stage Liver Disease; SD, standard deviation.

**Supplementary Table 4. Demographic and clinical characteristics of HEV patients with or without cirrhosis.**

| **Characteristic** | **HEV without cirrhosis (n=1639)** | **HEV with cirrhosis**  **(n=274)** | ***P* value** |
| --- | --- | --- | --- |
| Age, y, mean ± SD | 50.15±14.20 | 50.91±12.09 | 0.400 |
| Male sex | 1398(85.3) | 243(88.7) | 0.161 |
| ALB, g/L, mean ± SD | 36.09±8.63 | 32.84±6.58 | <0.001 |
| Missing, % | 91(5.6) | 9(3.3) |  |
| PLT, ×10^9^ cells/L, mean ± SD | 185.83±72.39 | 110.80±64.65 | <0.001 |
| Missing, % | 125(7.6) | 13(4.7) |  |
| ALT, U/L, median (IQR) | 502.00(122.25,1123.00) | 123.00(55.00,452.00) | <0.001 |
| Missing, % | 87(5.3) | 9(3.3) |  |
| T-BiL, μmol/L, median (IQR) | 116.55(39.73,220.50) | 207.30(73.05,367.48) | <0.001 |
| Missing, % | 87(5.3) | 10(3.6) |  |
| INR, mean ± SD | 1.17±0.63 | 1.64±0.94 | <0.001 |
| Missing, % | 136(8.3) | 14(5.1) |  |
| SCR, μmol/L, median (IQR) | 82.00(72.00,92.00) | 84.00(71.00,100.00) | 0.071 |
| Missing, % | 121(7.4) | 13(4.7) |  |
| MELD score, mean ± SD | 15.17±6.10 | 18.94±8.55 | <0.001 |
| Missing, % | 149(9.1) | 16(5.8) |  |
| Hospitalization days | 26.68±17.44 | 32.16±25.08 | <0.001 |
| Co-morbidity number | 1.56±1.41 | 3.90±1.60 | <0.001 |
| All-cause mortality | 31(1.9) | 18(6.6) | <0.001 |
| Liver-related mortality | 24(1.5) | 18(6.6) | <0.001 |

Abbreviations: HEV, hepatitis E virus; ALB, albumin; PLT, blood platelet level; ALT, alanine aminotransferase; T-BiL, total bilirubin; INR, international normalized ratio; SCR, serum creatinine; MELD, Model for End-Stage Liver Disease; SD, standard deviation.

**Supplementary Table 5.** Demographic and clinical characteristics of HEV patients with or without hepatic coma.

| **Characteristic** | **HEV without hepatic coma (n=1879)** | **HEV with hepatic coma (n=34)** | ***P* value** |
| --- | --- | --- | --- |
| Age, y, mean ± SD | 50.20±13.95 | 53.29±11.78 | 0.199 |
| Male sex | 1610(85.7) | 31(91.2) | 0.465 |
| ALB, g/L, mean ± SD | 35.71±8.47 | 30.33±3.77 | <0.001 |
| Missing, % | 99(5.3) | 1(2.9) |  |
| PLT, ×10^9^ cells/L, mean ± SD | 176.32±75.71 | 94.48±43.93 | <0.001 |
| Missing, % | 137(7.3) | 1(2.9) |  |
| ALT, U/L, median (IQR) | 417.50(104.00,1027.75) | 92.00(64.50,575.50) | 0.018 |
| Missing, % | 95(5.1) | 1(2.9) |  |
| T-BiL, μmol/L, median (IQR) | 122.80(41.70,238.58) | 337.65(188.40,471.40) | <0.001 |
| Missing, % | 95(5.1) | 2(5.9) |  |
| INR, mean ± SD | 1.22±0.68 | 2.42±1.04 | <0.001 |
| Missing, % | 148(7.8) | 2(5.9) |  |
| SCR, μmol/L, median (IQR) | 82.00(71.00,93.00) | 89.00(78.00,112.00) | 0.017 |
| Missing, % | 133(7.1) | 1(2.9) |  |
| MELD score, mean ± SD | 15.73±6.44 | 27.34±6.51 | <0.001 |
| Missing, % | 162(8.6) | 3(8.8) |  |
| Hospitalization days | 27.47±18.72 | 27.20±24.01 | 0.935 |
| Co-morbidity number | 1.85±1.62 | 4.44±1.64 | <0.001 |
| All-cause mortality | 36(1.9) | 13(38.2) | <0.001 |
| Liver-related mortality | 30(1.6) | 12(35.3) | <0.001 |

Abbreviations: HEV, hepatitis E virus; ALB, albumin; PLT, blood platelet level; ALT, alanine aminotransferase; T-BiL, total bilirubin; INR, international normalized ratio; SCR, serum creatinine; MELD, Model for End-Stage Liver Disease; SD, standard deviation.

**Supplementary Table 6. Demographic and clinical characteristics of HEV patients with or without hepatorenal syndrome.**

| **Characteristic** | **HEV without hepatorenal syndrome (n=1891)** | **HEV with hepatorenal syndrome (n=22)** | ***P* value** |
| --- | --- | --- | --- |
| Age, y, mean ± SD | 50.17±13.90 | 58.14±13.24 | 0.008 |
| Male sex | 1619(85.6) | 22(100.0) | 0.061 |
| ALB, g/L, mean ± SD | 35.67±8.44 | 30.03±5.34 | 0.005 |
| Missing, % | 96(5.1) | 4(18.2) |  |
| PLT, ×10^9^ cells/L, mean ± SD | 175.46±76.08 | 110.61±41.47 | <0.001 |
| Missing, % | 134(7.1) | 4(18.2) |  |
| ALT, U/L, median (IQR) | 415.00(102.00,1028.00) | 91.00(53.25,329.25) | 0.011 |
| Missing, % | 92(4.9) | 4(18.2) |  |
| T-BiL, μmol/L, median (IQR) | 123.75(41.85,239.33) | 452.80(251.22,592.38) | <0.001 |
| Missing, % | 93(4.9) | 4(18.2) |  |
| INR, mean ± SD | 1.23±0.68 | 2.57±1.39 | <0.001 |
| Missing, % | 146(7.7) | 4(18.2) |  |
| SCR, μmol/L, median (IQR) | 82.00(71.00,93.00) | 166.00(102.25,220.75) | <0.001 |
| Missing, % | 130(6.9) | 4(18.2) |  |
| MELD score, mean ± SD | 15.75±6.36 | 33.71±7.66 | <0.001 |
| Missing, % | 161(8.5) | 4(18.2) |  |
| Hospitalization days | 27.49±18.80 | 25.17±20.76 | 0.571 |
| Co-morbidity number | 1.86±1.63 | 4.59±1.76 | <0.001 |
| All-cause mortality | 40(2.1) | 9(40.9) | <0.001 |
| Liver-related mortality | 33(1.7) | 9(40.9) | <0.001 |

Abbreviations: HEV, hepatitis E virus; ALB, albumin; PLT, blood platelet level; ALT, alanine aminotransferase; T-BiL, total bilirubin; INR, international normalized ratio; SCR, serum creatinine; MELD, Model for End-Stage Liver Disease; SD, standard deviation.

**Supplementary Table 7. Demographic and clinical characteristics of HEV patients with or without renal insufficiency.**

| **Characteristic** | **HEV without renal insufficiency (n=1884)** | **HEV with renal insufficiency (n=29)** | ***P* value** |
| --- | --- | --- | --- |
| Age, y, mean ± SD | 50.08±13.89 | 61.48±10.61 | <0.001 |
| Male sex | 1613(85.6) | 28(96.6) | 0.110 |
| ALB, g/L, mean ± SD | 35.72±8.44 | 28.82±4.69 | <0.001 |
| Missing, % | 99(5.3) | 1(3.4) |  |
| PLT, ×10^9^ cells/L, mean ± SD | 175.29±76.13 | 144.42±67.36 | 0.033 |
| Missing, % | 137(7.3) | 1(3.4) |  |
| ALT, U/L, median (IQR) | 419.00(102.50,1029.00) | 113.50(53.75,336.00) | 0.004 |
| Missing, % | 95(5.0) | 1(3.4) |  |
| T-BiL, μmol/L, median (IQR) | 124.50(41.90,241.48) | 252.25(119.15,450.45) | 0.001 |
| Missing, % | 96(5.1) | 1(3.4) |  |
| INR, mean ± SD | 1.24±0.71 | 1.30±0.44 | 0.644 |
| Missing, % | 149(7.9) | 1(3.4) |  |
| SCR, μmol/L, median (IQR) | 82.00(71.00,92.00) | 143.50(106.25,234.50) | <0.001 |
| Missing, % | 133(7) | 1(3.4) |  |
| MELD score, mean ± SD | 15.80±6.56 | 23.85±5.52 | <0.001 |
| Missing, % | 164(8.7) | 1(3.4) |  |
| Hospitalization days | 27.37±18.68 | 33.32±26.23 | 0.090 |
| Co-morbidity number | 1.86±1.63 | 4.34±1.76 | <0.001 |
| All-cause mortality | 46(2.4) | 3(10.3) | 0.036 |
| Liver-related mortality | 39(2.1) | 3(10.3) | 0.024 |

Abbreviations: HEV, hepatitis E virus; ALB, albumin; PLT, blood platelet level; ALT, alanine aminotransferase; T-BiL, total bilirubin; INR, international normalized ratio; SCR, serum creatinine; MELD, Model for End-Stage Liver Disease; SD, standard deviation.

**Supplementary Table 8. Demographic and clinical characteristics of HEV patients with or without renal failure.**

| **Characteristic** | **HEV without renal failure (n=1902)** | **HEV with renal failure (n=11)** | ***P* value** |
| --- | --- | --- | --- |
| Age, y, mean ± SD | 50.24±13.92 | 53.00±15.11 | 0.512 |
| Male sex | 1631(85.8) | 10(90.9) | 0.625 |
| ALB, g/L, mean ± SD | 35.65±8.44 | 29.46±3.83 | 0.015 |
| Missing, % | 100(5.3) | 0(0.0) |  |
| PLT, ×10^9^ cells/L, mean ± SD | 175.18±75.99 | 113.53±68.36 | 0.007 |
| Missing, % | 138(7.3) | 0(0.0) |  |
| ALT, U/L, median (IQR) | 410.00(100.75,1021.25) | 115.00(52.00,2662.00) | 0.878 |
| Missing, % | 96(5.0) | 0(0.0) |  |
| T-BiL, μmol/L, median (IQR) | 125.40(41.90,243.00) | 304.30(89.00,411.90) | 0.014 |
| Missing, % | 97(5.1) | 0(0.0) |  |
| INR, mean ± SD | 1.24±0.70 | 1.94±0.92 | 0.001 |
| Missing, % | 150(7.9) | 0(0.0) |  |
| SCR, μmol/L, median (IQR) | 82.00(71.00,93.00) | 176.00(111.00,251.00) | <0.001 |
| Missing, % | 134(7.0) | 0(0.0) |  |
| MELD score, mean ± SD | 15.85±6.54 | 28.64±7.46 | <0.001 |
| Missing, % | 165(8.7) | 0(0.0) |  |
| Hospitalization days | 27.50±18.85 | 20.46±11.30 | 0.216 |
| Co-morbidity number | 1.88±1.65 | 4.09±1.51 | <0.001 |
| All-cause mortality | 43(2.3) | 6(54.5) | <0.001 |
| Liver-related mortality | 37(1.9) | 5(45.5) | <0.001 |

Abbreviations: HEV, hepatitis E virus; ALB, albumin; PLT, blood platelet level; ALT, alanine aminotransferase; T-BiL, total bilirubin; INR, international normalized ratio; SCR, serum creatinine; MELD, Model for End-Stage Liver Disease; SD, standard deviation.

**Supplementary Table 9. The effect of end-stage liver diseases on clinical outcomes of HBV/HEV patients.**

| **Characteristic** | **MELD score** | **Hospitalization days** | **Co-morbidity number** | **Mortality** |
| --- | --- | --- | --- | --- |
| HBV/HEV patients without ESLD (n=244) | 11.76±7.03 | 30.45±19.28 | 2.31±1.58 | 3(1.2) |
| HBV/HEV patients with one ESLD (n=121) | 16.10±8.57 | 32.59±23.95 | 4.22±1.89 | 4(3.3) |
| HBV/HEV patients with two ESLD (n=85) | 23.15±7.82 | 33.99±30.26 | 5.93±1.74 | 11(12.9) |
| HBV/HEV patients with three ESLD (n=20) | 27.15±6.95 | 27.39±23.44 | 7.90±2.38 | 5(25.0) |
| HBV/HEV patients with four ESLD (n=2) | 34.88 | 36.28±15.39 | 8.00 | 2(100.0) |
| HBV/HEV patients with five ESLD (n=0) | - | - | - | - |
| *P* value | <0.001 | 0.644 | <0.001 | <0.001 |

Abbreviations: HBV, hepatitis B virus; HEV, hepatitis E virus; ESLD, end-stage liver disease; MELD, Model for End-Stage Liver Disease.
